# LGR4 Is a Direct Target of MicroRNA-34a and Modulates the Proliferation and Migration of Retinal Pigment Epithelial ARPE-19 Cells

**DOI:** 10.1371/journal.pone.0168320

**Published:** 2016-12-15

**Authors:** Qiang Hou, Linglin Zhou, Jiajia Tang, Nan Ma, Ancong Xu, Jiang Tang, Dandan Zheng, Xiaogang Chen, Feng Chen, Xiang Da Dong, LiLi Tu

**Affiliations:** 1 School of Ophthalmology and Optometry, Eye Hospital, Wenzhou Medical University, Wenzhou, Zhejiang, China; 2 State Key Laboratory and Key Laboratory of Vision Science, Ministry of Health of the People’s Republic of China, Zhejiang Provincial Key Laboratory of Ophthalmology and Optometry, Wenzhou, Zhejiang, China; 3 Department of Surgery, Westchester Medical Center, Valhalla, New York, United States of America; Eye Hospital, Charité, GERMANY

## Abstract

The pathology of proliferative vitreoretinopathy and proliferative diabetic retinopathy is linked to proliferation, migration, and adhesion of the retinal pigment epithelium. MicroRNA-34a (miR-34a) expression modulates changes in proliferation and migration of retinal pigment epithelial cell line ARPE-19. In this study, we determined that miR-34a interacts with LGR4, identified by bioinformatics using TargetScan Human 5.0, to affect these changes. Double luciferase gene reporter assay confirmed miR-34a involvement in mediating control. miR-34a mimic transfection decreased LGR4 expression. Western blot analysis documented corresponding protein expression inhibition. MTS, Ki67 immunostaining, scratch and transwell testing, along with attachment assay showed that miR-34a upregulation inhibited ARPE-19 cell proliferation, migration and attachment partly through downregulation of LGR4 protein expression. Western blot analysis revealed that both miR-34a upregulation and LGR4 downregulation induced declines in E2F1, p-CDC2, CDK2, CDK4 and CDK6 protein expression. Taken together, miR-34a gene expression upregulation inhibits ARPE-19 cell proliferation, migration and adhesion partly by suppressing LGR4 expression. These results substantiate earlier indications that both miR-34a and LGR4 are potential drug targets to prevent fibrosis in a clinical setting.

## Introduction

MicroRNAs (miRNAs) are non-protein-coding, short (20–25) nucleotide RNAs that regulate expression of at least 30% of all genes.[1,2] MiRNA-protein complexes bind to the 3'-untranslated region of their target mRNAs, leading to inhibition of translation or destabilization of the target mRNAs.[2–4] miR-34a, a gene product of chromosomal locus 1p36.23, is a tumor suppressor in many types of tumors.[5] This miRNA is the focus of many research endeavors to better understand regulatory mechanisms of cell proliferation and migration. One of the findings showed that the miR-34 family are targets of the p53 oncogene family.[6] In mammals, the miR-34 family contains three processed miRNAs with miR-34a being ubiquitously expressed.[5] The other two, miR-34b and miR-34c share a common primary transcript and are mainly expressed in the lungs.[5] miR-34a expression is particularly pronounced in the CNS system and implicated in controlling cell proliferation, cell cycle arrest and senescence.[5,7] The identified target genes of miR-34a include silent information regulator 1 (SIRT1), Bcl-2, CD44, c-Met, various cyclins and CDKs, and the proto-oncoproteins MYC, MYCN amongst numerous others.[5,8]

The retinal pigment epithelium (RPE) is composed of a monolayer of hexagonal cells that are quiescent and do not undergo proliferation and migration under normal conditions. Following breaching of the blood retinal barrier by injury, RPE exposure to blood borne cytokines can cause the cells to differentiate into a fibroblastic phenotype. The proliferative and migratory changes are followed by expression of α-smooth muscle actin (α-SMA) contractile proteins leading to pathologic conditions including proliferative vitreoretinopathy (PVR), age-related macular degeneration and diabetic retinopathy.[9]

Altered miRNA expression affects target genes responsible for changes in the phenotype of ARPE-19 cell, a human retinal epithelial cell line. One of the miRNA changes includes variations in miR-124 expression which affect epithelial mesenchymal transition (EMT).[10] miR-124 was downregulated in transforming growth factor-β1 (TGF-β1) induced EMT process in retinal pigment epithelium. Furthermore, miR-124 inversely regulated TGF-β1-induced EMT in ARPE-19 cells.[10] In another study employing ARPE-19 cells, miR-29b also inversely regulated TGF-β1 and EMT and ARPE-19 transition.[11] We reported recently that miR-34a is one of the regulators highly expressed in post-confluent RPE cells. Its regulatory function was confirmed by showing that miR-34a upregulation inhibits RPE cell proliferation and migration through directly or indirectly suppressing the expression of c-Met and other cell cycle-related molecules.[8]

LGR4 (leucine rich repeat containing G protein-coupled receptor 4), also known as Gpr48 (G protein-coupled receptor 48), is a member of the G protein-coupled receptor (GPCR) family.[12–15] The role of LGR4 in enhancing the aggressiveness of carcinoma as well as developmental derangements such as intrauterine growth retardation or renal development is well documented.[16–19] It has also been verified as a prognostic factor for poor outcome in cancer patients.[20] In terms of ocular development, we demonstrated that knockout of LGR4 reduced epithelial cell proliferation and migration and resulted in EOB (eye open at birth) phenotype in mice. Because of failure in eyelid closure, the eyes of mice harboring EOB phenotype are open at birth unlike wildtype mice whose eyes are closed at birth but open by 12 to 14 days postpartum.[13,15] We also found that deletion of LGR4 in mice can cause anterior segment dysgenesis and onset of age-related cataracts.[12,14] There is, however, no report about the role of LGR4 in the regulation of RPE functional activity.

In this study, we demonstrated that LGR4 is a direct target of miR-34a. Inhibition of LGR4 expression with a specific siRNA suppressed ARPE-19's proliferative and migratory activity. Transfection with a miR-34a mimic or siRNA targeting LGR4 decreased ARPE-19 cell adhesion. Furthermore, miR-34a and LGR4 have similar downstream targets involved in mediating control of cell cycle progression. These include E2F1, p-CDC2, CDK2, CDK4 and CDK6. Taken together, our results suggest that LGR4 is a direct target of miR-34a and regulates proliferation, migration and adhesion of ARPE-19 cells.

## Materials and Methods

### Cell Culture

ARPE-19, a human retinal epithelial cell line, and human embryonic kidney 293 cells (HEK293) were purchased from American Type Culture Collection (ATCC; Manassas, VA). ARPE-19 cells were cultured in DMEM-F12 medium (Invitrogen, Carlsbad, CA) with 10% fetal bovine serum (FBS; Hyclone, Logan, UT) at 37°C in 5% CO_2_. HEK293 cells were cultured in DMEM (Invitrogen) with 10% FBS at 37°C in 5% CO_2_.

### Luciferase Reporter Assay

A 1.3 kb 3’-untranslated region (UTR) fragment of LGR4 containing the putative target site of miR-34a was subcloned into the SpeI-SacI sites of the dual luciferase pMIR-REPORT vector (Ambion, Austin, TX) with forward primer: 5’- CCACTAGTAAAGAACAGGTGCCTAAA and reverse primer: 5’-AAGGAGCTCAACGAAATGAACAGAAGC. For mismatch constructs, eight mismatches (underlined in the primers sequence) were introduced in the putative target site with QuikChange^®^ XL Mutagenesis Kit (Stratagene, La Jolla, CA) according to the manufacturer’s instruction. The primers used for this purpose are: forward 5’-CAGTGTGCTAAATCAATAGCAAACCACCGTCACTATTAGTTATTCTG and reverse 5’- CAGAATAACTAATAGTGACGGTGGTTTGCTATTGATTTAGCACACTG.

HEK293 is a cell line widely used to perform dual luciferase assay to investigate the activation/inhibition of many signal pathways such as Wnt, NF-κB, TGF-β, as well as the validation of direct interaction between miRNAs and their targets. In this study, HEK293 cells in 96-well plates were co-transfected with pRL-SV40 vector (Promega, Madison, WT), which contains *Renilla* luciferase, and pMIR-REPORT vector containing the firefly luciferase. For each transfection, 100 nM negative control miRNA (GenePharma, Shanghai, China) or a miR-34a mimic molecule (GenePharma) was co-transfected with the reporter constructs using Lipofectamine 2000 (Invitrogen) according to the manufacturer’s instructions. MiRNA mimics are small, double-stranded RNA molecules that mimic endogenous mature miRNA molecules when transfected into cells. In this study, we used a nonsense sequence as controls. Luciferase activity was analyzed 24 hours after transfection, using a dual-luciferase reporter assay system (Promega). Firefly luciferase activity was normalized to *Renilla* luciferase activity. All transfections were performed in 6-well duplicates in 96-well plates (Costar, High Wycombe, UK).

### Cell Proliferation Assay

2,000 ARPE-19 cells were seeded per well in 96-well plates 24 hours before transfection. LGR4 specific siRNA (5’- GGUAAGAAACUCCUAAUUAUU, GenePharma), negative control (NC) siRNA (GenePharma) or mock was used to downregulate LGR4 expression. For each well, LGR4 specific siRNA (100 nM), negative control siRNA (100 nM) or mock was transfected into ARPE-19 cells with Lipofectamine 2000 (Invitrogen). Cell proliferation was evaluated with the MTS assay after 72 hours culture according to the manufacturer’s instruction (Promega).

### Immunofluorescent Staining

ARPE-19 cells were transfected either with 100 nM miR-34a mimic, LGR4 siRNA, a negative control (NC) or mock. Forty-eight hours after transfection, cells were fixed with 4% formaldehyde in phosphate-buffered saline (PBS) for 20 minutes at room temperature, followed by permeabilization with 0.1% Triton X-100 in PBS for 20 minutes. After blocking with 1% bovine serum albumin (BSA) in PBS for 10 minutes, cells were incubated with primary antibody Ki67 diluted in PBS (1:1000, Millipore, Billerica, MA) overnight. Cy3-conjugated secondary antibody was incubated with the cells for 1 hour in the dark after rinsing with PBS. Cells were washed and then incubated for 10 minutes with 4’-6-Diamidino-2-phenylindole (DAPI) to monitor the nuclei. Cells were mounted and images were captured using a fluorescence microscope (Imager Z1, Zeiss, Oberkochen, Germany). Ki67 positive cells are expressed as percentages based on the number of Ki67 positive cells in three random visual fields divided by the number of DAPI positive cells.

### Transwell Migration Assays

100 nM LGR4 siRNA, NC or mock was introduced into ARPE-19 cells grown to approximately 60% confluence. After 48 hours, trypsin was used to detach the cells, which were washed once with D-Hank’s solution (Invitrogen). 2 x 10^4^ cells were added into the upper chambers of the transwell that is separated with the lower chambers by a 8-μm pore size culture insert (Transwell; Costar). 400 μL of DMEM-F12 containing 10% FBS were added into the lower chambers. The transwell was placed at 37°C in a 5% CO_2_ atmosphere to allow the cells to migrate into the lower chambers. After 18 hours, the migrated cells were stained with crystal violet and observed under microscope (Zeiss) using a 20X objective. To further quantify the migration potential, cells in 5 random vision fields were counted.

### In Vitro Scratch Assay

100 nM LGR4 siRNA, NC or mock was introduced into ARPE-19 cells at approximately 60% confluence. A 200 μL pipette tip was used to scratch the monolayer when the cells reached confluence after 48 hours. After removing floating cells with Hank’s medium, cells were then cultured in 2 mL fresh serum free medium. Photographs were taken after scratching at 48 and 72 hours of culture using a 10X objective (Imager Z1; Zeiss). Cells that migrated into the gap in 4 vision fields were counted to evaluate their migratory ability.

### Cell Attachment Assay

ARPE-19 cells were grown in DMEM-F12 containing 10% FBS to approximately 30% confluence and transfected with 100 nM LGR4 siRNA or a miR-34a mimic, a negative control or underwent a sham procedure. After 48 hours, cells were trypsinized, and 8,000 cells were seeded in 96-well plates for 2 hours. Unattached cells were discarded, and the attached cells were washed twice with PBS. The relative number of attached cells was assessed with the MTS assay.

### Western Blot Analysis

1 x 10^5^ ARPE-19 cells were seeded in 6-well plates and grown in DMEM-F12 with 10% FBS for 24 hours and then transfected with LGR4 siRNA, miR-34a or negative control. Cells lysates were harvested after 72 hours and proteins were detected as previously described.[8] CDK2, CDK4, CDK6, p-CDC2 (threonine 161) and E2F1 antibodies were purchased from Cell Signaling Technology (Beverly, MA). Antibodies for β-actin and LGR4 were from Santa Cruz Biotechnology (Dallas, TX) and Abcam (Cambridge, MA) respectively. All primary antibodies were used at a dilution of 1:1000 and the secondary antibodies were used at 1:5000. The bands density was quantified with ImageJ software (National Institutes of health, Bethesda, MD).

### Statistical Analysis

All data were shown as the mean ± SD. Statistical analysis was performed using the Student’s *t*-test. *p* < 0.05 was considered significant.

## Results

### LGR4 Is a Direct Target of miR-34a

**Fig 1:**
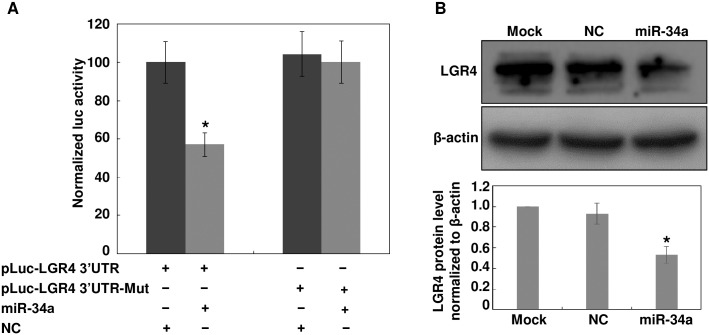
LGR4 is a direct target of miR-34a. (A) HEK293 cells were co-transfected with miR-34a, pMIR-LGR4 3’-UTR or pMIR-LGR4 3’-UTR-Mut, along with a pRL-SV40 reporter plasmid. After 24 hours, the luciferase activity was measured. Values are presented as relative luciferase activity after normalized to *Renilla* luciferase activity. Results are expressed as mean ± SD. n = 3, *p < 0.05. (B) ARPE-19 cells were transfected with either miR-34a or a negative control (NC). Cell lysates were prepared and used for Western blot analysis of LGR4. β-actin was probed as loading control. The band intensity was measured with ImageJ software. Images are representative of at least three independent experiments.

To predict potential miR-34a target genes, TargetScan (http://www.targetscan.org) was used. One region in the 3’-UTR of LGR4 was identified as a binding site for miR-34a. To confirm miR-34a directly targets LGR4, the wildtype 3’-UTR of LGR4 was cloned into a luciferase reporter vector. This vector was transfected into HEK293 cells along with miR-34a or a negative control (NC). Luciferase activity assays 24 hours after transfection demonstrated that miR-34a reduced luciferase activity to 57 ± 11% of the negative control transfection, whereas mutation of the target site abolished the reduction of luciferase activity induced by miR-34a (Fig 1A). To further validate that miR-34a directly regulates the expression of LGR4 in ARPE-19 cells, we performed Western blot analysis. LGR4 expression was significantly decreased 72 hours after transfection of miR-34a into ARPE-19 cells. The band density indicated a 53 ± 8% inhibition of LGR4 after introduction of miR-34a (Fig 1B). These data confirm that LGR4 is a direct target gene of miR-34a.

### LGR4 Downregulation Inhibits Cell Proliferation

**Fig 2:**
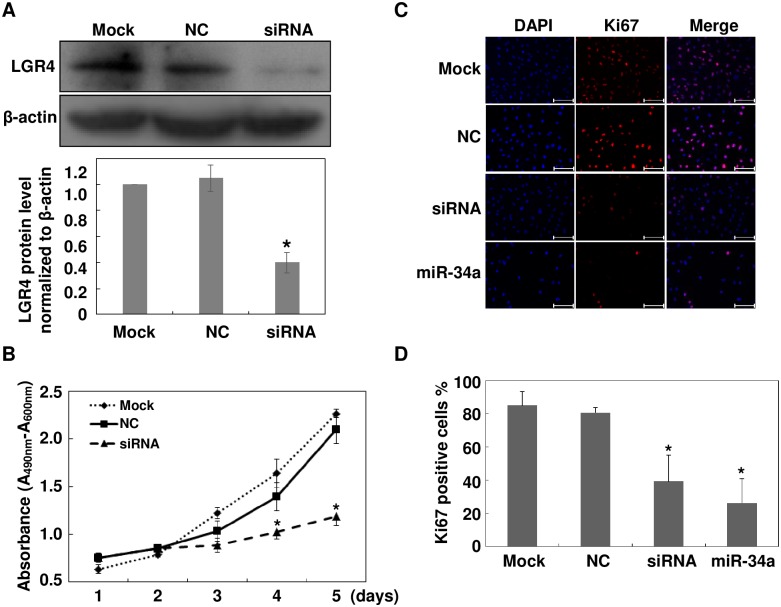
Knockdown of LGR4 inhibits ARPE-19 cell proliferation. Either a miR-34a mimic, LGR4 specific siRNA or a negative control (NC) was transfected into ARPE-19 cells. (A) Western blot analysis evaluated knockdown efficiency. The band intensity was measured with ImageJ software and normalized to the expression level in mock-treated cells. (B) MTS assay measured cell proliferation rates. Data at each time point were expressed as mean ± SD of the results obtained from three independent assays each performed in triplicates. (C) Ki67 immunostaining identified proliferating cells 48 hours after transfection. Images are representative of those obtained in three independent experiments. Scale bar: 200 μm. (D) Ki67 positive cells were expressed as percentages based on the number of Ki67 positive cells in three visual fields divided by the number of DAPI positive cells. Results are expressed as mean ± SD. n = 3, *p < 0.05.

As LGR4 overexpression promotes proliferation, migration and invasion of some cancer cell lines, we sought to determine if its downregulation can negatively impact ARPE-19 cells.[17] Transfection of ARPE-19 cells with a specific LGR4 siRNA significantly reduced LGR4 protein level after 72 hours (Fig 2A). Based on the MTS cell proliferation assay and Ki67 immunostaining results, LGR4 siRNA significantly inhibited growth of ARPE-19 cells three days after transfection (Fig 2B and 2C). Relative to the mock transfected group, cell proliferation was decreased to 40 ± 8% five days after being transfected with LGR4 siRNA. On the other hand, in the mock transfected group, proliferation remained the same. Similarly, the results shown in Fig 2C indicate that transfection of a LGR4 specific siRNA or a miR-34a mimic dramatically reduced ARPE-19 Ki67 staining. Ki67 positive cells were 39 ± 16% and 26 ± 15% in LGR4 siRNA and miR-34a transfected cells, respectively, whereas they were 85± 8% and 81 ± 3%, respectively, in the mock and NC groups (Fig 2D). These results show that there is a direct relationship between decreases in LGR4 expression and ARPE-19 cell proliferation.

### Downregulation of LGR4 Attenuates Cell Migratory Activity

**Fig 3:**
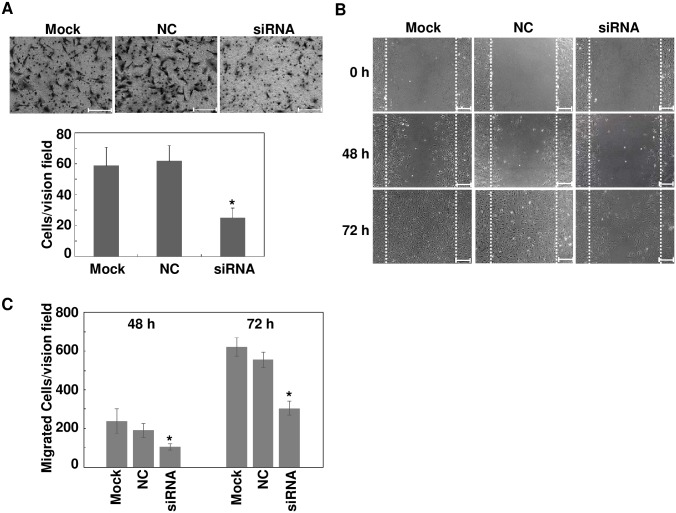
Downregulation of LGR4 decreases ARPE-19 cell migration. ARPE-19 cells were transfected with LGR4 specific siRNA or a scrambled negative control (NC). Transwell (A) and *in vitro* scratch assays (B) were performed to evaluate the ARPE-19 cell migration. The number of cells that migrated in the transwell assay was quantified by counting the number of cells appearing in the lower chamber in five independent vision fields with a 20X microscope objective. (C) For *in vitro* scratch assay, cells that migrated into the gap were counted at 48 hours and 72 hours as indicated. Results were expressed as mean ± SD (n = 3, *p < 0.05). All images are representative of at least three independent experiments. Scale bar: 100 μm for transwell assay, and 200 μm for *in vitro* scratch assay.

miR-34a inhibits both ARPE-19 cell proliferation and migration.[8] Since LGR4 is a target gene of miR-34a, we sought to confirm that LGR4 knockdown by itself has the same effect on ARPE-19. Accordingly, transwell and scratch wound assays were performed on LGR4 specific siRNA transfected ARPE-19 cells. In the transwell assay, much fewer LGR4 specific siRNA transfected cells migrated to the lower chamber (Mock: 59 ± 12, NC: 62 ± 10, siRNA: 25 ± 6 cells/vision field, n = 3, *p* < 0.05, Fig 3A). Similarly, fewer LGR4 specific siRNA transfected cells migrated into the gap between the leading edges of the wound compared with those appearing in plates containing mock transfected and NC cells (48 hours: Mock: 236 ± 64, NC: 191 ± 37, siRNA: 105 ± 15 cells/vision field; 72 hours: Mock: 621 ± 48, NC: 555 ± 39, siRNA: 303 ± 36 cells/vision field; n = 3, *p* < 0.05) (Fig 3B and 3C). Thus, downregulation of LGR4 inhibits migration of ARPE-19 cells.

### LGR4 Knockdown or miR-34a Ectopic Expression Decreases Cell Adhesion

**Fig 4:**
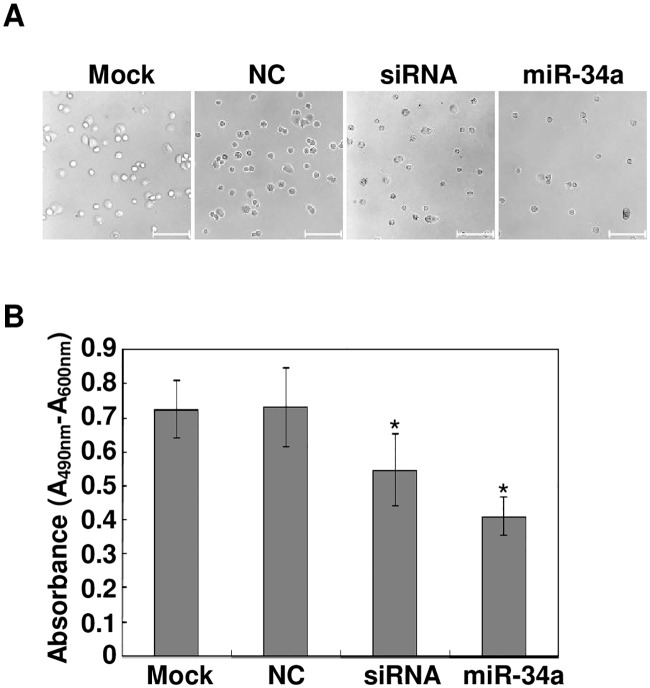
miR-34a expression or LGR4 knockdown attenuates ARPE-19 adhesion. Forty-eight hours after transfection with either a miR-34a, a LGR4 specific siRNA or a negative control (NC), ARPE-19 cells were harvested and re-seeded for 2 hours, and then unattached cells were removed by rinsing with PBS. (A) The phase-contrast images of attached cells were captured with an attached camera. Images represent those obtained in three independent experiments. Scale bar: 100 μm. (B) Attached cells were quantitated with the MTS assay. Data were expressed as mean ± SD of results from three independent experiments with each repeated in triplicates. *p < 0.05.

Stress-induced RPE cell detachment from Bruch’s Membrane is one of the initial steps in the pathogenesis of PVR. To determine if interactions of miR-34a with LGR4 affected ARPE-19 adhesion to its substratum, we compared the effects of LGR4 loss of function with those of miR-34a mimic transfection on this process. LGR4 knockdown and miR-34a mimic transfection both significantly decreased ARPE-19 cell attachment (Fig 4A). To quantitate these declines, the MTS assay was performed in parallel on these two groups of cells (Fig 4B). The absorbances at 490 nm for the siRNA group and miR-34a transfected cells were much lower compared with mock and NC groups (Mock: 0.72 ± 0.08, NC: 0.73 ± 0.12, siRNA: 0.55 ± 0.11, miR-34a: 0.41 ± 0.06). These results suggest that there is an inverse relationship between miR-34a expression and ARPE-19 adhesion since miR-34a induced suppression of LGR4 expression inhibited adhesion.

### Knockdown of LGR4 Downregulates the Expression of Cell Cycle-Related Mediators

**Fig 5:**
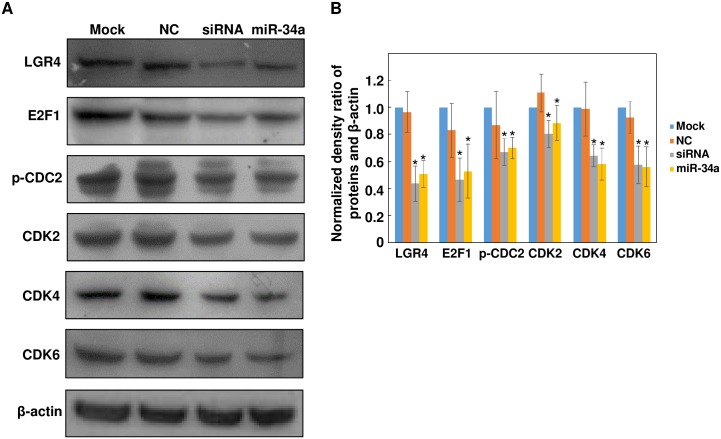
Knockdown of LGR4 downregulates the expression of cell cycle-related molecules reminiscent of miR-34a mimic transfection. (A) After transfection with either LGR4 specific siRNA or miR-34a mimic, ARPE-19 cell lysates were prepared and probed with anti-LGR4, E2F1, p-CDC2, CDK2, CDK4 and CDK6 antibodies. β-actin was probed as a loading control. These results are representative of at least three independent experiments. (B) The band intensity was measured with ImageJ software. The densitometric ratio between proteins of interest and β-actin was calculated and then normalized to the expression level in mock-treated cell. Results were expressed as mean ± SD (n = 3, *p < 0.05).

We further explored if direct knockdown of LGR4 had similar effects on the expression or activation of downstream effectors of miR-34a.[8] Western blot analysis showed that LGR4 siRNA transfection suppressed expression of E2F1, phosphorylated-CDC2 (p-CDC2), CDK2, CDK4 and CDK6 compared with mock or NC group, similar to effects caused by miR-34a mimic transfection (Fig 5A). The band density was quantified with ImageJ software and normalized to the level of β-actin (Fig 5B). These results are in accordance with others showing that increase in miR-34a expression suppresses proliferation by inhibiting LGR4 expression. This inhibitory effect of miR-34a on proliferation is consistent with the declines in the expression of the above-mentioned cell cycle mediators. There is, however, inconsistency in the extent to which these genes are inhibited by miR-34a as compared with our previous results,[8] especially for E2F1, p-CDC2 and CDK6.

## Discussion

Previously, we have shown that miR-34a modulates proliferation and migration of ARPE-19 cells through changes c-Met expression.[8] miR-34a upregulation suppressed c-Met expression which led to declines in ARPE-19 cell proliferation and migration. In the current study, LGR4 was identified as another direct target gene of miR-34a whose modulation controls ARPE-19 cell proliferation, migration and adhesion. As with c-Met, increases in miR-34a expression suppressed both LGR4 expression levels and ARPE-19 cell proliferation and migration. We further showed that miR-34a mimic transfection also inhibited attachment. This study again demonstrates that one specific miRNA can modulate cellular responses through control of numerous target genes.

The RPE forms part of the blood retinal barrier and serves multiple functions needed for normal vision. This quiescent monolayer reduces impinging retinal light intensity, maintains a homeostatic milieu for retinal function in the subretinal environment and re-isomerizes trans-retinal to 11-cis-retinal needed for photo transduction by the photoreceptors.[21] On the other hand, following breaching of the blood retinal barrier by injury, RPE cells can be exposed to blood borne cytokines and gain proliferative and migratory potential in pathological conditions.[9] RPE cells are the most important cells involved in the occurrence of PVR.[22] Uncontrolled proliferation and migration of RPE cells eventually form pathologic membranes on both surfaces of the neural retina that can contract and cause retinal detachment and visual impairment.[22]

In this study, we used the ARPE-19 cell line to investigate the potential role of LGR4 in RPE cells. ARPE-19 cells express the retinal pigment epithelium-specific 65 kD protein (RPE65) and cellular retinaldehyde-binding protein (CRALBP). They are also the most popular cell line in RPE cell research.[23] Numerous studies use ARPE-19 cell line as a model to analyze the molecular and genomic properties of RPE under pathological conditions, in order to develop new approaches to treatment of ophthalmological diseases.[24] To the best of our knowledge, this study is the first one to present a functional role LGR4 in ARPE-19 cells.

LGR4 is widely expressed in multiple organs such as heart, lung, intestines, kidneys, cartilage, reproductive tracts and the nervous system.[25] It is essential for normal embryonic development because studies with knockout mice identified a wide variety of developmental defects.[26] Specifically, LGR4 is normally expressed in a spatiotemporal pattern in the developing eye. By E12.5, LGR4 is primarily expressed in a layer of mesenchymal cells between the ocular surface and the optic cup.[14] Neonatal mice retain strong expression in the ciliary body, iris stroma, lens and the corneal endothelial and epithelial cells.[14] In addition to the embryonic lethality previously shown, LGR4^-/-^ mice had multiple ocular defects including microphthalmia, partial corneal opacity, anterior segment dysgenesis, keratopathies, eye open at birth and early onset cataract formation.[14] LGR4^-/-^ keratinocytes also showed decreased migration and proliferation as a result of EGFR inactivation.[13,15]

After confirming LGR4 is a direct target of miR-34a, we found that LGR4 knockdown inhibited ARPE-19 cell proliferation, migration and adhesion. miR-34a mimic transfection also inhibited these responses. Taken together, these results show that the described inverse relationship between changes in miR-34a and c-Met expression also pertains to the effects of variations in miR-34a on LGR4 expression.

Recent studies have shown that LGR4 is highly expressed in several types of cancer including gastric, lung, prostate and colorectal cancer.[17,20] p27^Kip1^ induced LGR4 upregulation can enhance colon cancer progression and metastasis.[19] LGR4 promotes prostate tumorigenesis through PI3K/Akt signaling pathway.[16] Hsu et al. identified a LGR4 splice variant which encodes a soluble secreted protein that can inhibit LGR4 mediated Wnt signaling.[27] Furthermore, a nonsense mutation of LGR4 (c.376C>T) has been reported to be strongly associated with low bone marrow density, electrolyte imbalance, reduced testosterone levels and increased risk of squamous cell carcinoma of the skin.[28] Therefore, identifying regulators of LGR4 expression and activity may have a significant impact in clinical practice.

In summary, LGR4 is a direct target gene of miR-34a. miR-34a upregulation had inverse effects on LGR4 expression as well as inhibiting ARPE-19 proliferation, migration and adhesion. miR-34a and LGR4 are potential targets for drug development for treating retinal fibrosis since either miR-34a mimic transfection or LGR4 siRNA downregulation suppressed ARPE-19 cell proliferation, migration and adhesion.
